# Progressive and concordant alterations in transcriptional and gut microbiota across aortic valve calcification severity

**DOI:** 10.1128/spectrum.02137-25

**Published:** 2025-12-22

**Authors:** Jue Wang, Ruihang Qu, Wenhao Huang, Yue Chen, Yun Li, Qingqing Lin, Ziji Wu, Hangfei Yan, Tingting Yu, Chiyin Wang, Xinlei Ren, Xiaobing Wang, Jinyu Wu

**Affiliations:** 1 Department of Cardiac Surgery, First Affiliated Hospital of Wenzhou Medical University 89657, Wenzhou, China; 2 Key Laboratory of Laboratory Medicine, Ministry of Education, Institute of Genomic Medicine, Wenzhou Medical University 26453, Wenzhou, Zhejiang, China; 3 Department of Rheumatology and Immunology, First Affiliated Hospital of Wenzhou Medical University 89657, Wenzhou, China; 4 Department of Rheumatology and Immunology, Changzheng Hospital, Naval Medical University 56652, Shanghai, China; China Agricultural University, Beijing, China

**Keywords:** aortic valve calcification, gut microbiota, transcriptional profiles, functional analysis

## Abstract

Calcific aortic valve disease (CAVD) is a common disorder associated with substantial morbidity and mortality. Although the gut microbiome has complex associations with cardiovascular disease, its variation across the calcification spectrum in CAVD remains poorly defined. We profiled aortic-valve transcriptomes from 31 patients spanning graded calcification and paired these with matched stool microbiome profiles. We identified subtle yet widespread transcriptional changes in mild CAVD (m-CAVD), consistent with a progressive relationship between calcification burden and gene-expression remodeling. At the community level, the gut microbiome in m-CAVD exhibited an intermediate configuration between non- and higher-calcification profiles, suggesting an early shift in the gut ecosystem along the disease continuum. At the genus level, we identified 11 taxa associated with stage; notably, *Anaerococcus* increased with calcification burden, whereas *Rheinheimera* declined across stages. These results refine the pathophysiology landscape of CAVD by connecting stage-dependent valvular transcriptional changes with coordinated shifts in the gut microbiome and indicate that early, microbiome-targeted interventions may be promising.

**IMPORTANCE:**

Calcific aortic valve disease is a common valvular heart disease. Due to the difficulty in sampling arterial calcified tissues, research on the interaction between their gene expression and the gut has been limited. In this study, by analyzing the transcriptional profiles of calcified aortic valve tissues from patients with different levels of calcification and the characteristics of their corresponding gut microbiota, we identified consistent features between lesion gene expression and gut microbiota variation. This provides important evidence for the association between the gut microbiota and disease development stages, offering a new perspective for understanding disease progression and early intervention.

## INTRODUCTION

Calcific aortic valve disease (CAVD) is one of the most prevalent heart valve disorders, characterized by the thickening of the valve and the formation of calcified nodules, often accompanied by osteogenic differentiation and neovascularization within the valvular tissue (1–7). Beyond classical cardiovascular risk factors (e.g., hypertension, hyperlipidemia, and diabetes), accumulating evidence suggests that gut microbiome dysbiosis may influence cardiovascular susceptibility through immune, metabolic, and atherogenic pathways (2, 8–10).

The gut microbiota is a complex consortium of bacteria, fungi, and viruses that contributes to host metabolism, immune modulation, and the maintenance of intestinal barrier integrity (11, 12). Emerging data link between dysbiosis and CAVD onset and progression. Patients with CAVD display microbial community structures and functions that differ from those in coronary artery disease (13). Microbial metabolites engage in complex interactions with distant organs and signal systemically. For example, gut bacteria convert dietary choline and carnitine into trimethylamine, which is subsequently oxidized into trimethylamine N-oxide (TMAO) in the liver. TMAO has immune-modulating and pro-inflammatory activities and is associated with endothelial cell damage and elevated cardiovascular risk (14, 15). Dysbiosis is also accompanied by metabolic derangements, including increased cholesterol metabolites and shifts in short-chain fatty acids (SCFA) profiles, which have been implicated in valvular calcification (16–18).

In parallel, disruption of the intestinal barrier can permit the translocation of endotoxins and other microbe-associated molecular patterns, amplifying systemic inflammation (19–21). Such signals can activate valvular endothelial and interstitial cells, promoting osteogenic programs and apoptosis that may accelerate calcification (22). Together, these observations motivate a focused evaluation of microbiome-valve interactions in CAVD.

Despite growing links between the gut microbiota and CAVD, stage-resolved features across the calcification spectrum remain undefined, partly due to limited access to human valve tissue. We therefore integrated aortic valve transcriptomes across graded calcification with matched stool microbiomes to relate stage-dependent gene programs to community, taxonomic, and functional shifts, providing a framework for early microbiome-informed risk stratification and therapy.

## MATERIALS AND METHODS

### Study population and sample collection

Patients with CAVD who underwent aortic valve replacement were registered in the First Affiliated Hospital of Wenzhou Medical University. We excluded patients with acute infection of inflammatory bowel disease or autoimmune disease and valvular endocarditis. Immediately after resection, the calcific aortic valve tissue was divided into two parts: one was fresh for immunoassay, and the other was frozen (−80°C) until DNA was extracted.

Based on the calcification score (calcification score = Agatston score × computed tomography [CT] value), the samples were classified into three groups: severe calcification (severe CAVD [s-CAVD], *n* = 7), mild calcification (mild CAVD [m-CAVD], *n* = 16), and non-calcification (*n* = 8). The classification thresholds were as follows: mild 400–1300 for females and 1,000–2,000 for males; severe >1,300 for females and >2,000 for males (23).

### RNA sequencing of aortic valve tissue

According to the manufacturer’s protocol, total RNA was extracted from valve tissue using TRIzol Reagent (Thermo Fisher). The NEBNext Ultra RNA Library Prep Kit for Illumina (NEB, USA, Cat. #E7530L) was utilized to generate sequencing libraries from total RNA as the starting material, following the recommended procedures, with unique index codes incorporated into the adapter sequences of each sample. mRNA was enriched from total RNA using oligo(dT)-conjugated magnetic beads.

In the NEBNext First Strand Synthesis Reaction Buffer (5×), template RNA is denatured in the presence of divalent cations under elevated temperature. First-strand cDNA synthesis is performed using random hexamer primers and RNase H-deficient M-MuLV reverse transcriptase, followed by second-strand synthesis employing DNA Polymerase I and RNase H. The resultant DNA overhangs are converted to blunt ends via exonuclease/polymerase activity. Following 3′-end adenylation of DNA fragments, NEBNext Adaptors (hairpin loop structure) are ligated to facilitate hybridization. Library fragments are size-selected (370–420 bp) using the AMPure XP system (Beverly, USA). Subsequently, 3 µL of USER Enzyme (NEB, USA) is added for fragment processing, with incubation at 37°C for 15 min and heat inactivation at 95°C for 5 min. PCR amplification is then conducted using Phusion High-Fidelity DNA Polymerase with Universal PCR Primers and Index (X) Primer. The PCR products are purified via AMPure XP beads, and library quality is evaluated on an Agilent 5400 system (Agilent Technologies, USA). Quantitative PCR (qPCR; 1.5 nM) is employed for precise quantification. Finally, qualified libraries are pooled according to effective concentration and target data output, followed by Illumina platform sequencing (PE150 strategy) at Novogene Bioinformatics Technology Co., Ltd. (Beijing, China).

### Collection of fecal samples and 16S rRNA gene sequencing

Fecal samples were obtained from 30 adult volunteers. Samples were collected using rectal swabs, stored in a −80°C freezer, and then transported to the testing center. Extract 1,000 µL of CTAB lysis buffer into a 2.0 mL Eppendorf tube, add lysozyme, introduce an appropriate amount of sample into the lysis buffer, and incubate at 65°C with periodic inversion to ensure thorough sample lysis. Centrifuge to collect the supernatant, add a mixture of phenol (pH 8.0):chloroform:isoamyl alcohol (25:24:1), invert and mix, and centrifuge at 12,000 rpm for 10 min. Transfer the supernatant, add chloroform:isoamyl alcohol (24:1), invert and mix, and centrifuge at 12,000 rpm for 10 min. Transfer the supernatant to a 1.5 mL centrifuge tube, add isopropanol, shake gently, and precipitate at −20°C. Centrifuge at 12,000 rpm for 10 min and decant the liquid without disturbing the precipitate. Wash twice with 1 mL of 75% ethanol; the remaining small amount of liquid can be collected by centrifugation and pipetted out. Dry the workstation or air-dry at room temperature.

DNA samples are dissolved in nuclease-free ddH_2_O, with optional incubation at 55°C–60°C for 10 min to facilitate complete dissolution. RNA digestion is performed using 1 µL RNase A (10 mg/mL) at 37°C for 15 min, followed by the assessment of DNA purity and integrity through 1% agarose gel electrophoresis. DNA quantification is carried out using the Qubit dsDNA HS Assay Kit on a Qubit 2.0 Fluorometer (Thermo Fisher Scientific, CA, USA). An appropriate aliquot is transferred to a nuclease-free microcentrifuge tube and diluted with molecular-grade water to achieve an optical density (OD₂₆₀/₂₈₀) between 1.8 and 2.0. For library preparation, 1 µg of genomic DNA is fragmented to approximately 350 bp using a Covaris S220 ultrasonicator (duty cycle: 10%; intensity: 5; 60 s treatment time), followed by library construction with the NEBNext Ultra II DNA Library Prep Kit (NEB, USA) according to the manufacturer’s protocol, including end repair, dA-tailing, adapter ligation, size selection, and PCR amplification. Following library construction, initial quantification is performed using Qubit 2.0, with subsequent dilution to 2 ng/µL. Library fragment size distribution is verified using an Agilent 2100 Bioanalyzer with High Sensitivity DNA chips, followed by qPCR (KAPA Library Quantification Kit) to determine effective library concentration (>3 nM). Qualified libraries are pooled in equimolar ratios based on effective concentrations and subjected to 150 bp paired-end sequencing on the Illumina platform (PE150).

### Bioinformatics analysis

DADA2 is a plugin used for sequence quality filtering, denoising, merging, and dereplication. It filters data by removing low-quality reads. Specifically, if the average quality score is below 20 and the length of the truncated read is reduced by more than 75% compared to the original read length, the entire sequence will be discarded. Additionally, reads containing primers/adapters and those with ambiguous bases (*N*) will also be removed to obtain high-quality clean data. After quality control, the raw data are archived, and optimized sequences are identified. Subsequently, based on a similarity threshold of ≥97%, original sequences are assigned to operational taxonomic units (OTUs) using UPARSE. Each OTU represents distinct taxonomic levels, including domain, phylum, class, order, family, and genus. Classification of each 16S rRNA gene sequence is performed by comparing the Ribosomal Database Project classifier algorithm with the Silva (SSU123) 16S rRNA database at a 70% confidence threshold. Bioinformatics analysis is conducted using QIIME (version 1.9.1) to assess the diversity of gut microbial communities through alpha diversity metrics (including Chao1 richness estimator, Shannon index, and inverse Simpson index) and beta diversity. Beta diversity analysis includes principal coordinates analysis and non-metric multidimensional scaling for visualizing structural variations in microbial communities between different groups. Furthermore, to detect differences in community structure (at the phylum and genus levels) between sample groups, linear discriminant analysis effect size (LEfSe) is employed to compare differences at the taxonomic level.

### Functional enrichment analysis

Gene Ontology (GO) function and Kyoto Encyclopedia of Genes and Genomes pathway enrichment analyses of the candidate differentially expressed genes (DEGs) were performed through clusterProfiler package. Through the application of the hypergeometric test based on the GO terms database, we discovered a number of significantly enriched GO terms and pathways. Enrichment was measured as the ratio of the DEGs to the genes in the entire genome, within the same GO term or pathway.

## RESULTS

### Analysis of patient valve tissue and stool samples

We conducted a retrospective study on 31 patients who underwent surgical treatment for aortic atherosclerosis at the First Affiliated Hospital of Wenzhou Medical University between 2021 and 2022. Patients were classified as severe calcification (s-CAVD, *n* = 7), mild calcification (m-CAVD, *n* = 16), or non-calcification (non-CAVD, *n* = 8) based on the calcification score (23). There were no significant between-group differences in age, sex, hyperlipidemia, hypertension, diabetes mellitus, or smoking status (Table S2).

**Fig 1:**
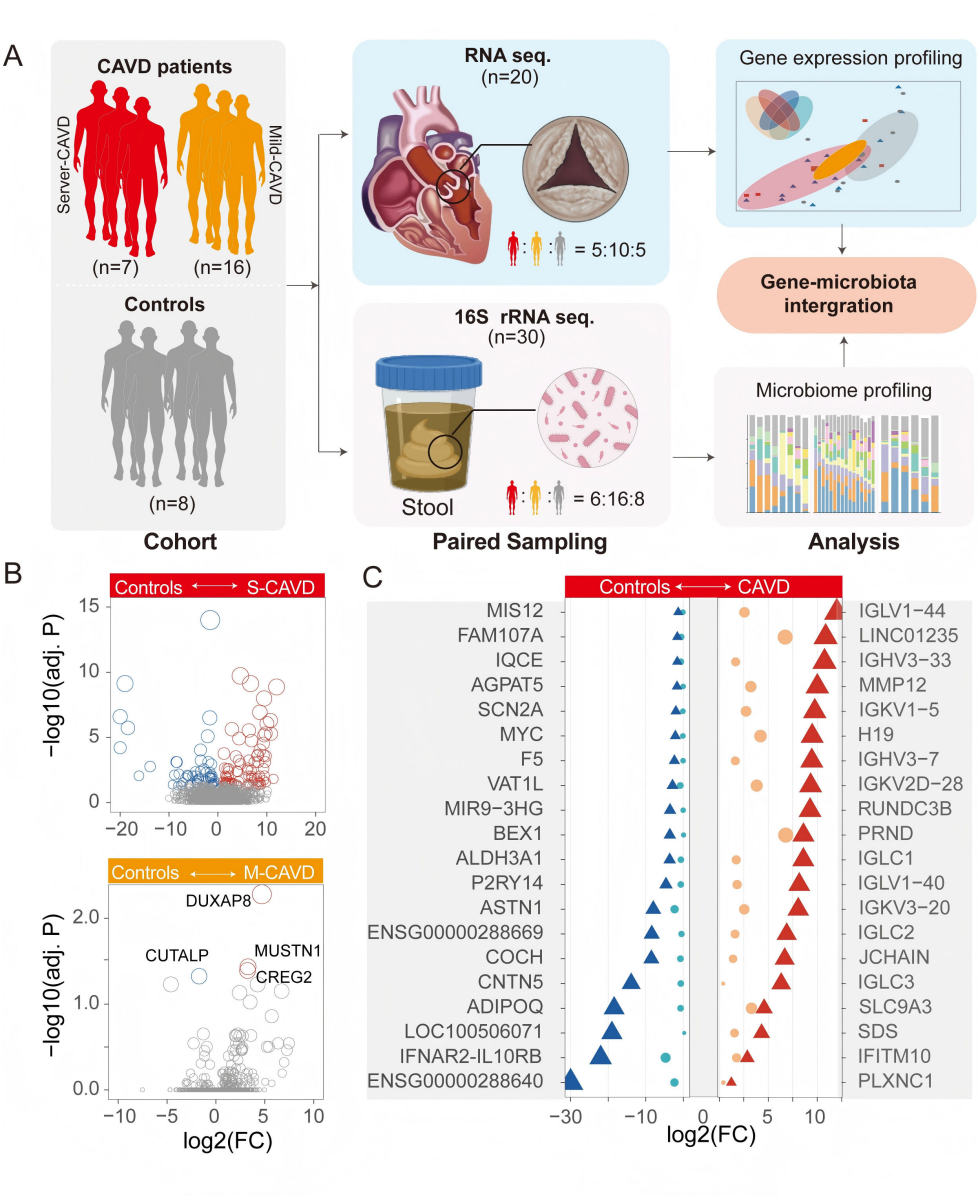
Study design and tissue DEG analysis in CAVD patients of varying severity and non-calcification controls. (**A**) Study design and sample collection for investigating gene expression and microbiome profiles in CAVD patients. Patients were divided into s-CAVD (*n* = 7) and m-CAVD (*n* = 16) groups, with an additional control group (*n* = 8) without CAVD. Paired sampling was performed for gene and microbiome analyses. RNA-seq was conducted on aortic valve tissue samples from 20 individuals (5 controls, 10 m-CAVD, and 5 s-CAVD), while 16S rRNA sequencing was conducted on stool samples from 30 individuals (8 controls, 16 m-CAVD, and 6 s-CAVD). (**B**) DEG analysis between control and CAVD groups: s-CAVD has 80 upregulated and 52 downregulated genes (top panel); m-CAVD has three upregulated and one downregulated gene (bottom panel). Thresholds for DEGs: adjusted *P* < 0.05, |logFC| > 0.5. *P*-values were calculated using a negative binomial model with the Wald test as implemented in DESeq2 based on input read counts, and adjusted *P*-values were computed using the Benjamini-Hochberg method to control the false discovery rate (FDR). (**C**) Top 20 DEGs with significant upregulation (red) or downregulation (blue) in s-CAVD compared to controls. Triangular points represent genes in the s-CAVD vs control comparison, while circular points represent genes in the m-CAVD vs control comparison. point size indicates the absolute value of the fold change.

RNA sequencing (RNA-seq) of aortic-valve tissue was performed for 20 participants (s-CAVD, *n* = 5; m-CAVD, *n* = 10; non-CAVD, *n* = 5) to profile gene expression. Additionally, fecal samples from 30 participants (s-CAVD, *n* = 6; m-CAVD, *n* = 16; non-CAVD, *n* = 8) underwent 16S rRNA gene sequencing to characterize the gut microbiota (Fig. 1A).

### Subtle yet widespread transcriptional alterations have already emerged in m-CAVD

To characterize transcriptional profiles associated with varying levels of cardiac valve calcification, we analyzed DEG across groups with different calcification severities and the non-calcification group. Compared to the non-calcification group, s-CAVD showed upregulation of 80 genes and downregulation of 52 genes (DEGs, *P* < 0.05, |logFC| > 0.5). In contrast, m-CAVD exhibited upregulation of only three genes (DUXAP8, MUSTN1, and CREG2) and downregulation of one gene (CUTALP). The s-CAVD group exhibited more DEGs than the m-CAVD group, consistent with broader transcriptional alterations in severe disease.

In s-CAVD, the most significantly upregulated genes included MMP12, IGHV3-33, LINC01235, IGLV1-44, RUNDC3B, IGKV2D-28, IGHV3-7, H19, and IGKV1-5. Among downregulated genes, ENSG00000288669, ENSG00000288640, IFNAR2-IL10RB, and LOC100506071 showed significant expression differences, though their roles remain largely uncharacterized due to their recent identification. These genes also exhibit similar directional changes in m-CAVD as in s-CAVD, though to a lesser extent (Fig. 1C).

**Fig 2:**
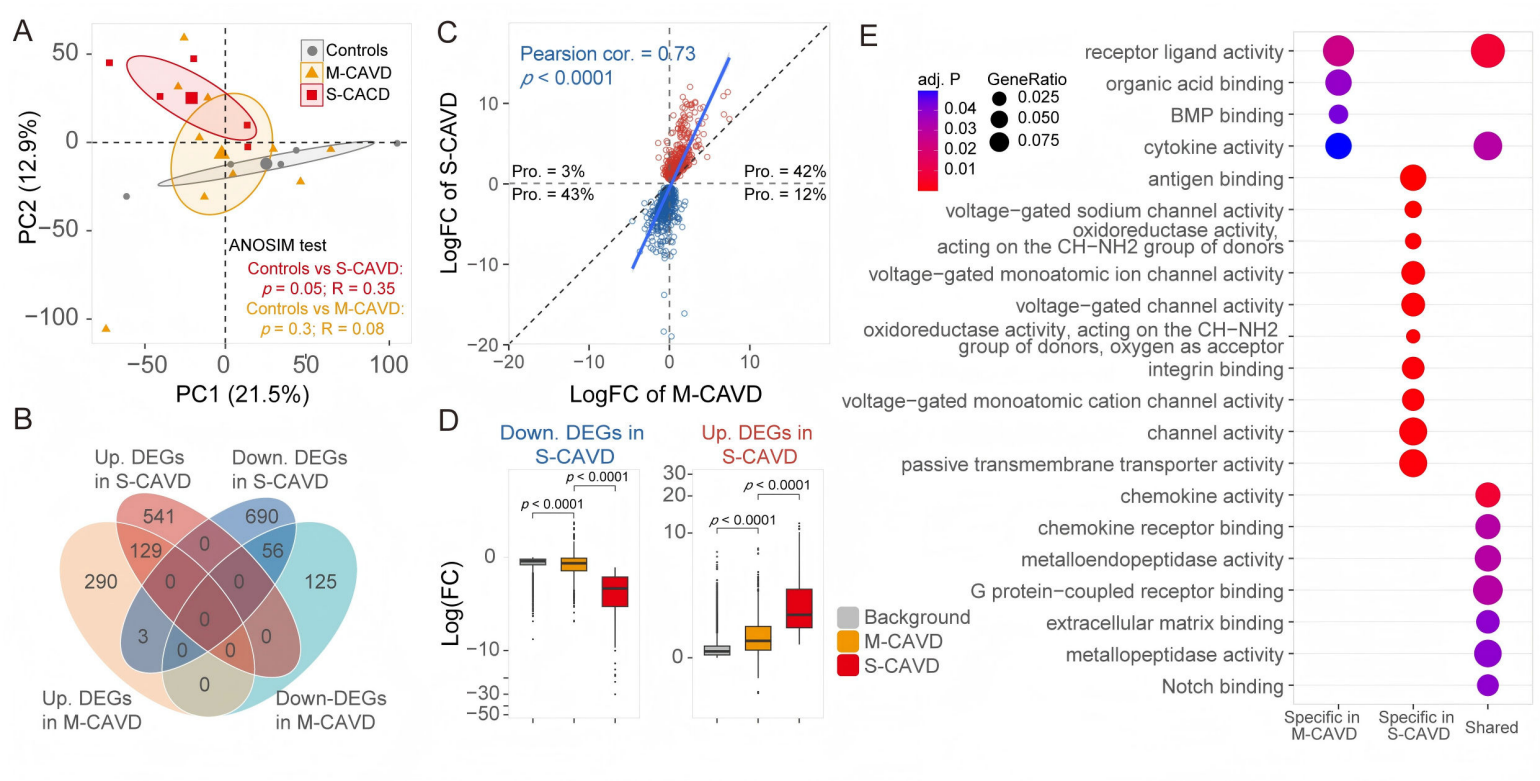
Emergence of subtle yet widespread transcriptional alterations in m-CAVD. (**A**) PCA was performed using the top 5,000 highly variable genes across all samples to explore overall transcriptional variance. Statistical significance of distribution differences among groups was assessed using the analysis of similarity (ANOSIM) test. The control group refers to the non-calcification group. (**B**) Venn diagram illustrating the overlap of DEGs across groups. (**C**) Expression patterns of DEGs (*n* = 1,419) identified in the s-CAVD vs control comparison, mapped onto the m-CAVD vs control group. Proportions of genes in each quadrant are labeled. (**D**) Boxplot showing the fold changes of 1,419 DEGs in m-CAVD (yellow) and s-CAVD (red) groups relative to the control group. Log fold changes of all other genes (excluding the 1,419 DEGs) in the m-CAVD vs control group are presented as a background reference (gray). The box represents the interquartile range (IQR) of the fold changes, with the line inside the box indicating the median value. Whiskers extend to the maximum and minimum values within 1.5 times the IQR from the quartiles, while points outside this range are plotted as individual outliers. *P*-values were calculated using a two-sided Mann-Whitney *U* test. (**E**) GO enrichment analysis results for DEGs across groups, highlighting significantly enriched functional categories. *P*-values for GO enrichment were calculated using a hypergeometric test to assess the over-representation of GO terms, and adjusted *P*-values were computed using the Benjamini-Hochberg method. A threshold of *P* < 0.05 was applied for DEG selection in analyses shown in panels **B**, **C**, **D**, and **E**.

To visualize severity-related gene expression patterns, we performed principal component analysis (PCA) based on highly variable genes among all samples. m-CAVD samples localized between the non-calcification and s-CAVD groups, with significant separation between non-calcification and s-CAVD groups (*P* = 0.05) but not between non-calcification and m-CAVD groups (*P* = 0.3; Fig. 2A). Next, we applied a more relaxed threshold for differential-expression analysis (unadjusted *P* < 0.05) to broaden signal detection. We identified 603 candidate genes in m-CAVD and 1,419 in s-CAVD. Despite a limited intersection (*n* = 188), 98.4% of the shared genes changed in the same direction; only three genes were downregulated in the s-CAVD but upregulated in the m-CAVD (Fig. 2B). Fold change profiles for the 1,419 s-CAVD genes correlated with those in m-CAVD (Pearson cor. = 0.73), with 85% showing concordant directionality.

The results of GO enrichment analysis indicate that genes differentially expressed solely in m-CAVD are enriched in the functions of organic acid binding and bone morphogenetic protein (BMP) binding. Particularly noteworthy is the enrichment of the BMP binding function, which has been previously validated in research linking BMP proteins to CAVD (24). On the other hand, genes exclusively enriched in the s-CAVD primarily encompass functions related to neural transmission, immune response, cellular structure and motility, cell signaling, and cell-cell interactions. Genes showing differential expression in both groups are enriched in processes related to cell structure, signaling, cellular functions, and tissue formation, including collagen-containing extracellular matrix, monocyte chemotaxis, and ossification (Fig. 2E; Fig. S1).

### Altered gut microbiota in m-CAVD represents a transitional state between non-calcification and severity

**Fig 3:**
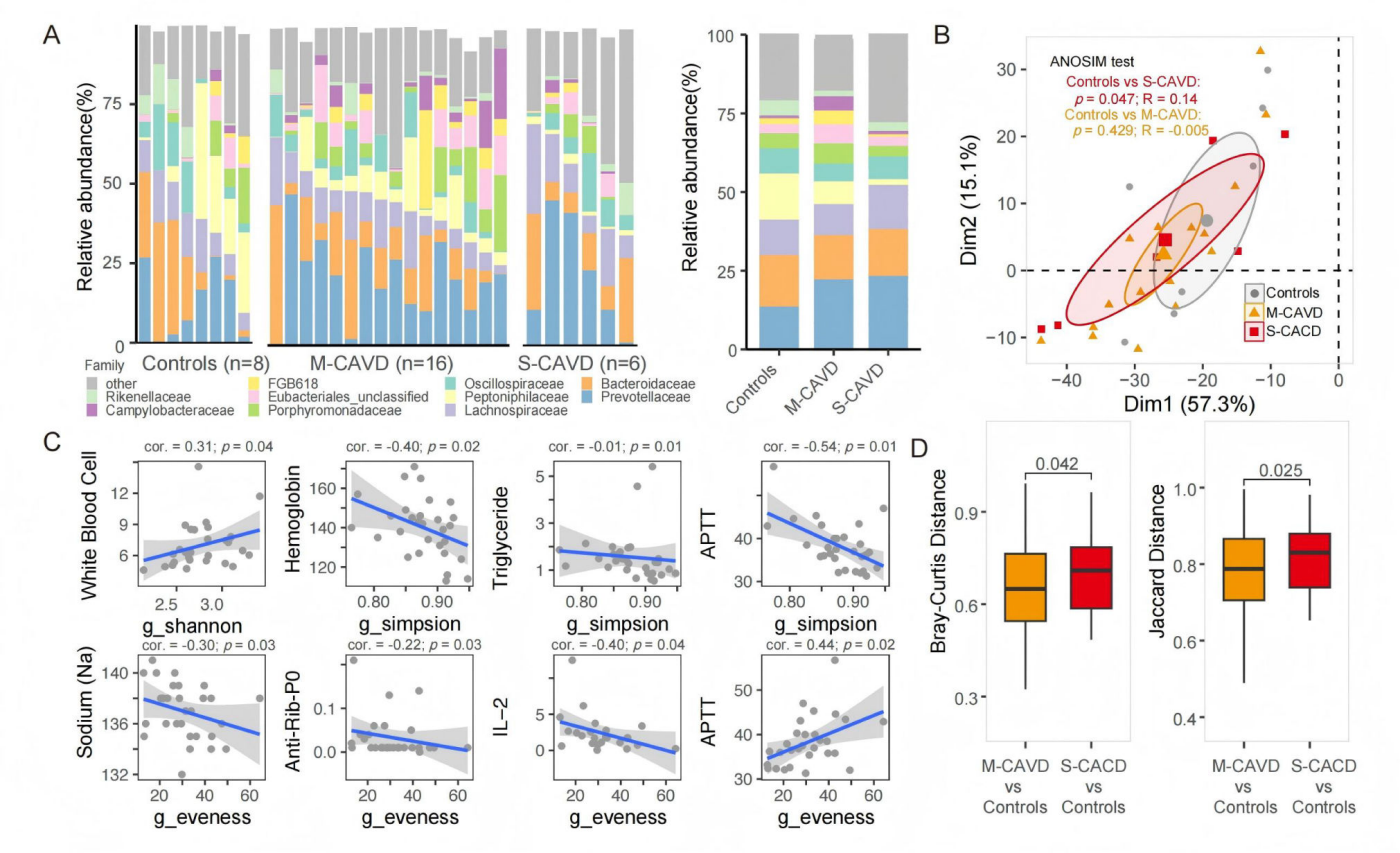
Gut microbiota was associated with different CAVD severity. (**A**) Relative abundance of gut microbiota at the family level across different samples. (**B**) PCA of patient gut microbiota at the genus level, with statistical significance assessed using the ANOSIM based on Bray-Curtis distances. (**C**) Pearson correlation analysis between alpha diversity metrics and clinical parameters. (**D**) Boxplot comparing Bray-Curtis distances (left) and Jaccard distances of samples from different severity levels of CAVD relative to the normal group. Statistical significance was determined using one-tailed *t*-tests. The sample sizes for the left and right boxes are 128 and 48, respectively. The box represents the interquartile range (IQR) of the fold changes, with the line inside the box indicating the median value. Whiskers extend to the maximum and minimum values within 1.5 times the IQR from the quartiles, while points outside this range are plotted as individual outliers. cor., Spearman correlation coefficient; p, *P*-value.

Several studies have linked gut microbiota to cardiovascular disease risk (2, 8, 11, 12). However, its role in cardiac valve calcification remains unclear. To investigate the gut microbiota characteristics in patients with differing degrees of calcification, we performed saturated 16S rRNA gene sequencing on 30 individual fecal samples (Fig. S2). The predominant bacterial families identified in the three groups were *Prevotellaceae*, *Bacteroidaceae*, and *Lachnospiraceae. Prevotellaceae* exhibited slightly higher proportions in both the severe and m-CAVD than controls (Fig. 3A). Conversely, *Peptoniphilaceae* was notably enriched in the non-calcification group, with its abundance significantly reduced in mild and s-CAVD, especially in the latter. Additionally, *Eubacteriales*_unclassified and *Campylobacteraceae* had lower levels in both the non-calcification and s-CAVD, while increased in the m-CAVD group (Fig. 3A).

We first observed that PCA positioned the m-CAVD samples between the s-CAVD and non-CAVD centroids. Consistent with this ordination pattern, pairwise analysis of similarity detected a significant difference between s-CAVD and non-CAVD (*R* = 0.14, *P* < 0.05), whereas comparisons involving m-CAVD were not significant (*P* = 0.429). Moreover, analyses based on Bray-Curtis and Jaccard distances corroborated these results, indicating greater dissimilarity between s-CAVD and non-CAVD than between m-CAVD and non-CAVD. Collectively, these results indicate that, at the community level, the microbial composition of m-CAVD is intermediate between the severe and non-calcified states (Fig. 3D).

Some studies suggest that inflammatory responses and abnormal immune reactions may play a role in the development of aortic valve calcification (25, 26). Therefore, we analyzed the relationship between certain inflammatory response and immune response markers and the richness of the microbiota. The Shannon index revealed a positive correlation between white blood cell levels and microbiota richness. In terms of the Simpson and evenness indices, levels of hemoglobin, triglycerides, anti-Rib-P0, IL-2, and sodium were all negatively correlated. However, there were differing patterns in the activated partial thromboplastin time indices (APTT indices; negative correlation in Simpson and positive correlation in evenness; Fig. 3C).

### Gut microbiota associated with calcification progression

**Fig 4:**
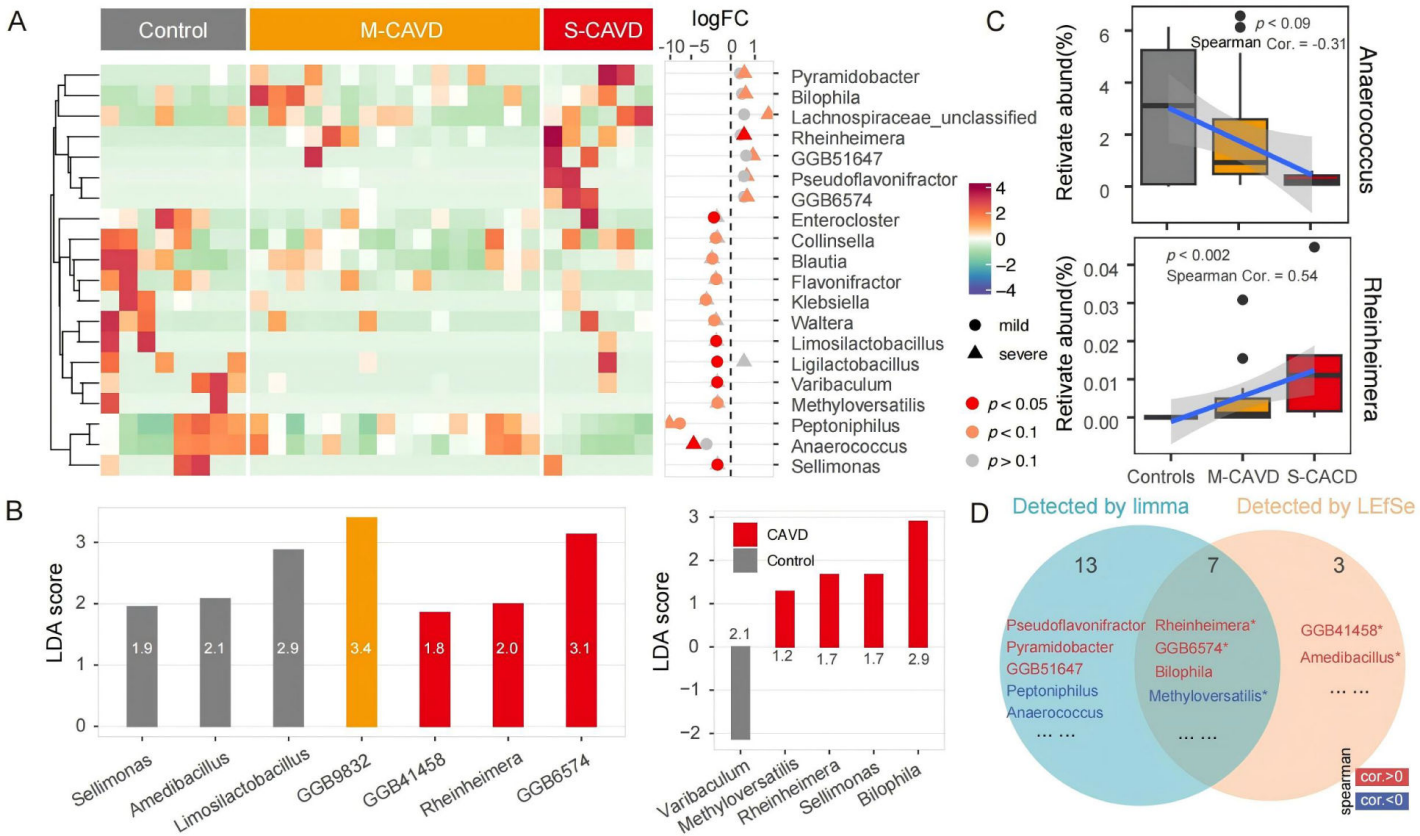
Differential analysis of gut microbiota across different groups. (**A**) Heatmap displaying the distribution of 29 potential differentially abundant gut microorganisms (genus level) identified using the limma package, with nine identified in the s-CAVD group and 12 in the m-CAVD group (*P* < 0.1). (**B**) Bar plot illustrating potential marker microorganisms identified through LEfSe analysis. (**C**) Spearman correlation analysis assessing the relationship between microbial abundance and disease severity. The box represents the interquartile range (IQR) of the fold changes, with the line inside the box indicating the median value. Whiskers extend to the maximum and minimum values within 1.5 times the IQR from the quartiles, while points outside this range are plotted as individual outliers. The sample sizes (*N*) for each group are as follows: controls (*n* = 8), m-CAVD (*n* = 16), and s-CAVD (*n* = 6). *P*-values were calculated using a *t*-test as implemented in cor. test to evaluate the significance of the correlation coefficient. (**D**) Venn diagram showing potential disease-associated microorganisms identified by different methods. The 11 labeled microorganisms exhibited Spearman correlations with disease severity, with *P*-values less than 0.1. Among these, four microorganisms with *P*-values less than 0.05 are indicated with asterisks.

To identify gut microbiota that may be involved in calcification processes, we utilized the limma package to assess differential bacteria between groups. Given our limited sample size, we adopted a more relaxed threshold (*P* < 0.1) and identified a total of 20 genera potentially associated with the degree of calcification (Fig. 4A). Among these, in the m-CAVD, the abundance of 12 bacteria was reduced compared to the non-calcification group (notably *Enterocloster*, *Limosilactobacillus*, *Ligilactobacillus*, *Varibaculum*, and *Sellimonas*, with the most significant differences at *P* < 0.05; the rest include *Methyloversatilis*, *Peptoniphilus*, *Collinsella*, *Blautia*, *Flavonifractor*, *Klebsiella*, *Waltera*). In the s-CAVD, there were nine differentially abundant bacteria, with seven exhibiting increased abundance (including *Pyramidobacter*, *Bilophila*, *Lachnospiraceae*_unclassified, *Rheinheimera*, GGB51647, *Pseudoflavonifractor*, GGB6574) and two showing decreased abundance (including *Peptoniphilus*, *Anaerococcus*). The abundance of *Peptoniphilus* remained consistent across both groups.

To identify marker bacterial taxa among the three groups, we employed the LEfSe method. In the non-calcification group, the marker taxa included *Sellimonas*, *Amedibacillus*, and *Limosilactobacillus*. The m-CAVD group was characterized by the unique genus GGB9832, while the marker bacteria for the s-CAVD group included GGB41458, *Rheinheimera*, and GGB6574 (Fig. 4B). When combining the m-CAVD and s-CAVD groups, *Bilophila* and *Varibaculum* were identified as marker taxa for the CAVD and control groups, respectively. Overall, the two differential analysis methods identified 23 distinct bacteria, with seven taxa supported by both approaches (Fig. 4D).

We performed a correlation analysis to assess the relationship between the abundance of these 23 potentially differential bacteria and the degree of calcification. This analysis showed a potential correlation (correlation *P*-value less than 0.1) for 78.2% of the examined bacteria (Fig. S3; Table S1), suggesting that alterations in the gut microbiota in m-CAVD may also be in a transitional state between non-calcification and severe conditions. Notably, we observed a decrease in *Anaerococcus* abundance across the three groups (*P* = 0.09) and an increase in *Rheinheimera* abundance (*P* = 0.002; Fig. 4C). Among the seven bacteria identified by both methods, *Rheinheimera*, GGB6574, and *Bilophila* exhibited potential positive correlations, while *Methyloversatilis* displayed a potential negative correlation (Fig. 4D).

### Functional analysis of genes associated with bacterial taxa

To delineate potential gut-valve regulatory pathways, 11 candidate functional bacterial taxa supported by ≥2 methods (limma, LEfSe, and Spearman correlation) were selected (Fig. 4D). For each taxon, we identified genes showing strong association with its abundance using Spearman correlation (*P* < 0.01, |cor.| > 0.2), and we then restricted candidates to those differentially expressed in the s-CAVD group (unadjusted *P* < 0.05, |logFC| > 0.5). This two-step procedure yielded 178 concordant genes and 265 discordant genes (Data S1). Concordant genes were positively correlated with taxa that increase with severity and negatively correlated with taxa that decrease with severity (i.e., gene-taxon effects aligned with the severity trend), whereas discordant genes showed the opposite pattern. Functionally, the concordant set likely marks pathways that intensify with calcification burden, while the discordant set may reflect compensatory or protective responses.

**Fig 5:**
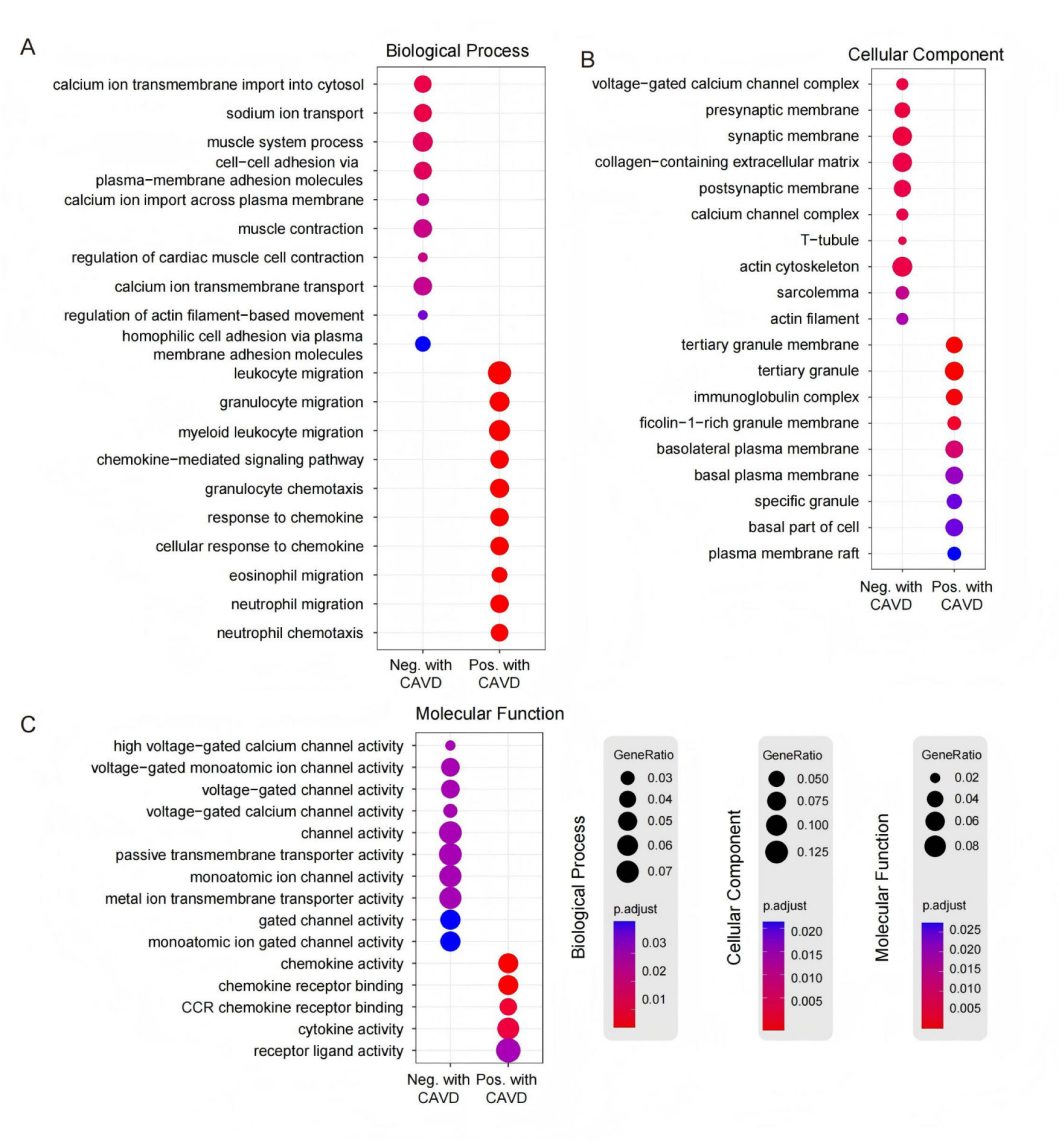
Functional enrichment analysis of genes associated with gut microbiota. (**A**) Biological process (BP) enrichment results. (**B**) Cellular component (CC) enrichment results. (**C**) Molecular function (MF) enrichment results. *P*-values for GO enrichment were calculated using a hypergeometric test to assess the over-representation of GO terms, and adjusted *P*-values were computed using the Benjamini-Hochberg method.

To infer putative microbial mechanisms, we performed GO enrichment analysis, which revealed that the concordant genes were enriched for immune and chemotactic programs, including leukocyte migration, granulocyte migration, cytokine activity, and chemokine receptor binding. These signals indicate heightened inflammatory/immune activation and cell trafficking, consistent with axes that could exacerbate calcification burden (Fig. 5). By contrast, the discordant genes were enriched for processes supporting cardiac pump function and tissue integrity—notably regulation of cardiac muscle cell contraction, calcium ion imports into the cytosol, and ion transmembrane transport—and they localized to structural compartments such as the actin cytoskeleton, synaptic membrane, and collagen-containing extracellular matrix (Fig. 5). Together, these patterns suggest protective/homeostatic axes. While hypothesis-generating rather than causal, the two sets delineate candidate gut-valve regulatory pathways for future mechanistic testing.

Enrichment analysis was conducted on the genes associated with the 11 bacteria individually (Fig. S4). Genes related to *Amedibacillus*, GGB41458, GGB6574, and *Pseudoflavonifractor* were found to be associated with polysaccharide binding (*P* < 0.04). Genes related to *Anaerococcus* were linked to the actin cytoskeleton (*P* < 0.02) and the voltage-gated calcium channel complex. GGB51647 and *Pyramidobacter* genes were associated with leukocyte migration (*P* < 0.02). *Methyloversatilis* genes were related to muscle contraction (*P* < 0.02), while *Peptoniphilus* genes were associated with leukocyte-specific tertiary granules (*P* < 0.02). The genes related to *Rheinheimera* were associated with functions related to energy conversion, membrane protein complexes, and intracellular ATP generation, such as the mitochondria proton-transporting ATP synthase complex and proton-transporting ATP synthase complex (*P* < 0.02; Fig. S4). The results of the enrichment analysis indicated that the genes associated with these bacteria are involved in various biological activities, including cell signaling, cell adhesion, ion channel functions, gene expression regulation, and muscle physiology.

## DISCUSSION

CAVD is a chronic, progressive valvulopathy characterized by leaflet thickening and non-rheumatic calcium deposition (27). Susceptibility is influenced by conventional risk factors such as hypertension, hyperlipidemia, and diabetes, alongside genetic and microenvironmental determinants. However, the mechanisms that drive stage-to-stage progression remain incompletely defined, particularly for gut-related pathways. While gut microbiota has been implicated in other cardiovascular conditions (13, 28). Unresolved issues in CAVD include population-level microbiome profiles, early microbial readouts, and causal links to disease biology. To address these gaps, we integrated valve RNA-seq with fecal 16S rRNA gene sequencing in patients spanning graded calcification.

Within severely calcified valves, we observed upregulation of MMP12, H19, and IGHV3-33, consistent with a multifaceted immuno-inflammatory signature. MMP12, a macrophage-derived metalloproteinase, promotes extracellular-matrix proteolysis and can potentiate osteogenic reprogramming of valvular interstitial cells, facilitating ectopic mineralization (29). The lncRNA H19 has been linked to epigenetic control of inflammatory and calcification pathways (30). Over-expression of IGHV3-33 suggests antigen-driven B-cell receptor activation and clonal immunoglobulin expansion, implicating adaptive immunity in valvular pathology. Together with prior evidence that cytokine signaling (e.g., TNF-α, IL-6, IL-1β) triggers osteogenic differentiation and that matrix metalloproteinases (MMPs, such as MMP-2/-9/-12) remodel matrix under inflammatory conditions (31–34), our data support an inflammation-to-calcification axis and motivate exploration of anti-inflammatory strategies.

Our findings align with large-scale valve transcriptomic studies that emphasized inflammatory signaling, matrix remodeling, and osteogenic programs in advanced CAVD (34) and with integrative work linking immune activation and metabolic reprogramming to calcification (24). Extending this literature, we show that these alterations emerge subtly in m-CAVD and intensify with severity, indicating a progressive continuum rather than discrete states. This stage-graded view refines current models by highlighting an early transitional state of gene expression that may be actionable for risk stratification and timely intervention.

Microbiome comparisons between calcified and non-calcified states indicate compositional shifts in the gut community. Notably, *Anaerococcus* shows a severity-related decline across groups, and prior work links this genus to trimethylamine (TMA) generation and atherosclerosis risk (35). This raises the hypothesis that reduced *Anaerococcus* may perturb cholesterol/TMA-TMAO-related metabolism and, in turn, influence CAVD progression, an idea that warrants targeted validation. We also observe *Collinsella* depletion in mild disease, whereas enrichment of this genus has been reported in symptomatic atherosclerosis (36). This discrepancy could reflect stage, population, medication, or sampling differences. Conversely, *Klebsiella* is decreased in our disease group despite associations with hypertension in other cohorts (37), again suggesting context dependence. Overall, these taxa are better viewed as entry points into metabolic-inflammatory pathways rather than stand-alone biomarkers; prospective studies integrating targeted metabolomics (e.g., TMA/TMAO, bile acids) and longitudinal sampling will be required to establish directionality and clinical relevance.

Recent studies converge on a role for gut dysbiosis in CAVD. Neiroukh et al. (38) reported reductions in short-chain-fatty-acid producers with enrichment of pro-inflammatory taxa, and Chong-Nguyen et al.^39^ described lower diversity with increases in taxa linked to metabolic dysfunction. Consistent with these reports, our data also point to inflammatory and metabolic axes. Extending this literature, we observe that microbial shifts are subtle in m-CAVD and more apparent in severe disease, suggesting a stage-graded pattern. By integrating metagenomes with valve transcriptomes, we further relate community changes to host immune-inflammatory programs (e.g., MMP12 and immunoglobulin variable genes), providing a complementary view of the microbiome-valve interface.

Preclinical work indicates that modulating the gut microbiota can ameliorate cardiovascular risk phenotypes: probiotic supplementation and SCFA delivery reduce atherosclerotic burden, attenuate systemic inflammation, and lower blood pressure in animal intervention studies (40, 41). Our stage-resolved data in m-CAVD and s-CAVD are consistent with this concept. They suggest that microbiome-directed strategies aimed at restoring SCFA-linked and other homeostatic pathways, or dampening pro-inflammatory signaling, could modify upstream risk factors relevant to CAVD and thereby offer a path to early-stage intervention. Prospective, mechanism-anchored trials that pair targeted microbial or metabolite interventions with valve imaging and immune/transcriptomic measures will be essential to determine efficacy and clinical utility.

## Data Availability

The raw sequencing data supporting the findings of this study have been deposited in the National Genomics Data Center (NGDC) under BioProject accession number PRJCA039760. This data set includes transcriptomic profiles of aortic valve tissues and 16S rRNA gene sequencing data of gut microbiota from 31 patients with varying degrees of aortic valve calcification. All data have undergone quality control and are publicly accessible.

## SUPPLEMENTAL MATERIAL

Data S1 spectrum.02137-25-s0001.xlsx Genes associated with the 11 candidate functional genera.

Supplemental material spectrum.02137-25-s0002.docx Fig. S1 to S4; Tables S1 and S2.

ASM does not own the copyrights to Supplemental Material that may be linked to, or accessed through, an article. The authors have granted ASM a non-exclusive, world-wide license to publish the Supplemental Material files. Please contact the corresponding author directly for reuse.
