# Supplementary material for: Progressive and concordant alterations in transcriptional and gut microbiota across aortic valve calcification severity
Source: Microbiol Spectr. 2025 Dec 22;14(2):e02137-25. doi: 10.1128/spectrum.02137-25 (PMC12889152; doi:10.1128/spectrum.02137-25)
Supplement: Supplemental material — Fig. S1 to S4; Tables S1 and S2. [file spectrum.02137-25-s0002.docx]

Supplementary Materials for

**Investigating the Relationship Between Gut Microbiota and Transcriptional Changes in Varying Degrees of Aortic Valve Calcification**

Jue Wang¹^, ^^, Ruihang Qu²^, ^^, Wenhao Huang³, Yue Chen³, Yun Li², Qingqing Lin³, Ziji Wu³, Hangfei Yan³, Tingting Yu³, Chiyin Wang³, Xinlei Ren¹, Xiaobing Wang³^, *^, Jinyu Wu^2, *^

**This PDF file includes:**

Figs. S1 to S4

Tables S1 to S2

**Figure S1.**


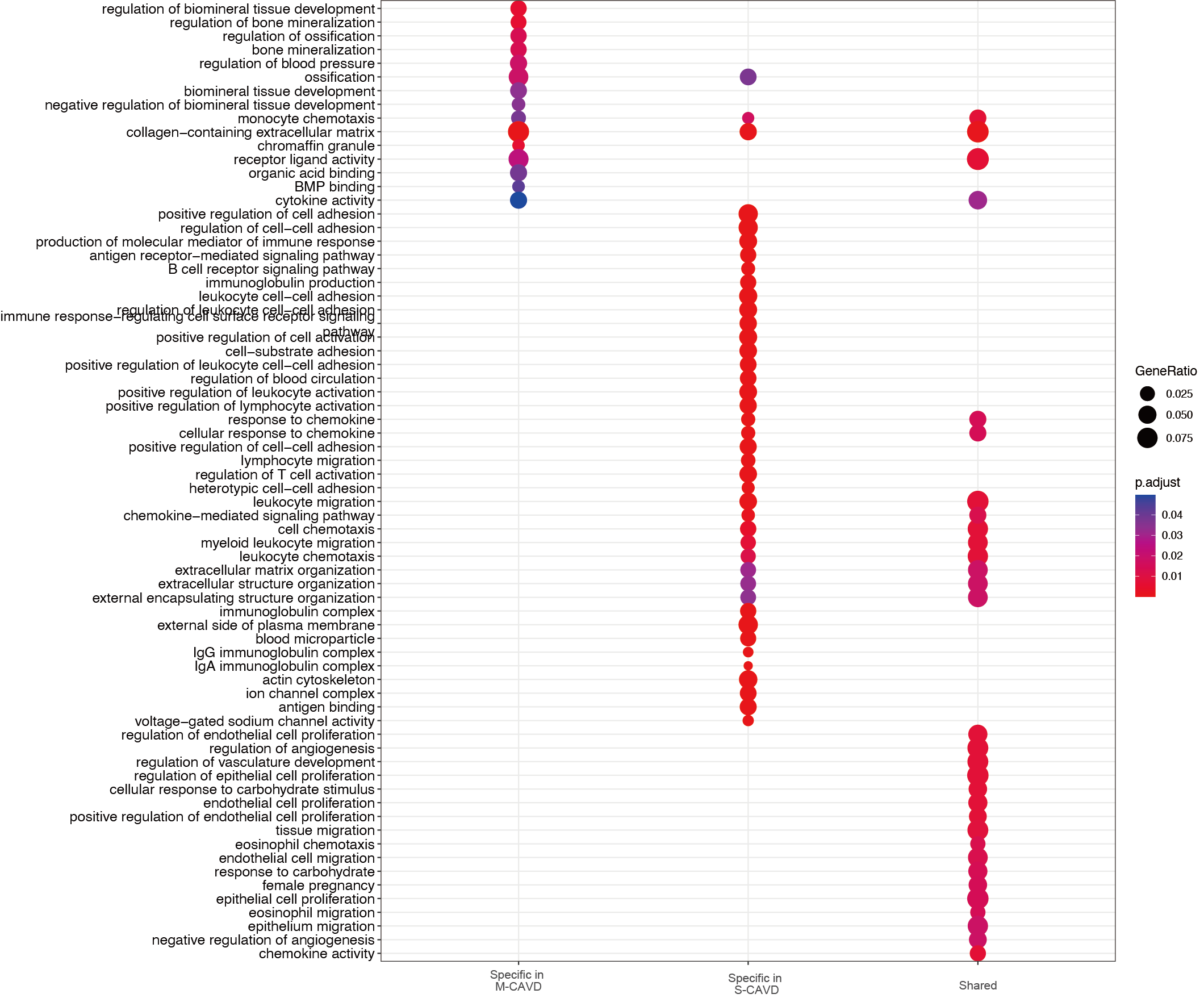


**Figure S1.** Comprehensive results of GO enrichment analysis, utilizing the same gene set as in Figure 2E. P-values for GO enrichment were calculated using a hypergeometric test to assess the over-representation of gene ontology terms, and adjusted p-values were computed using the Benjamini-Hochberg method.

**Figure S2.**


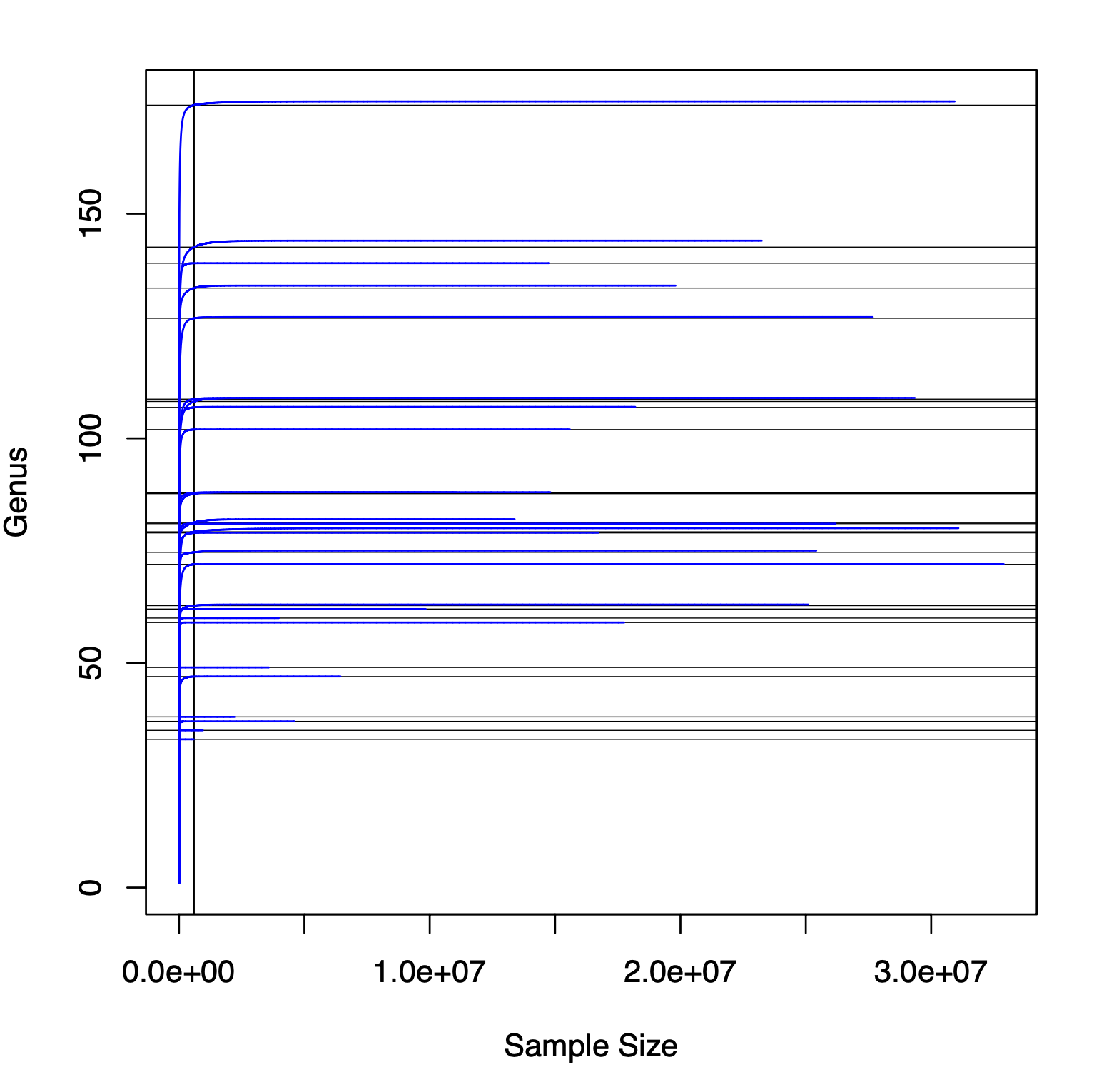


**Figure S2. Saturation curves of 16S rRNA sequencing for 30 fecal samples, illustrating the sequencing depth and the extent of genus captured.**

**Figure S3.**


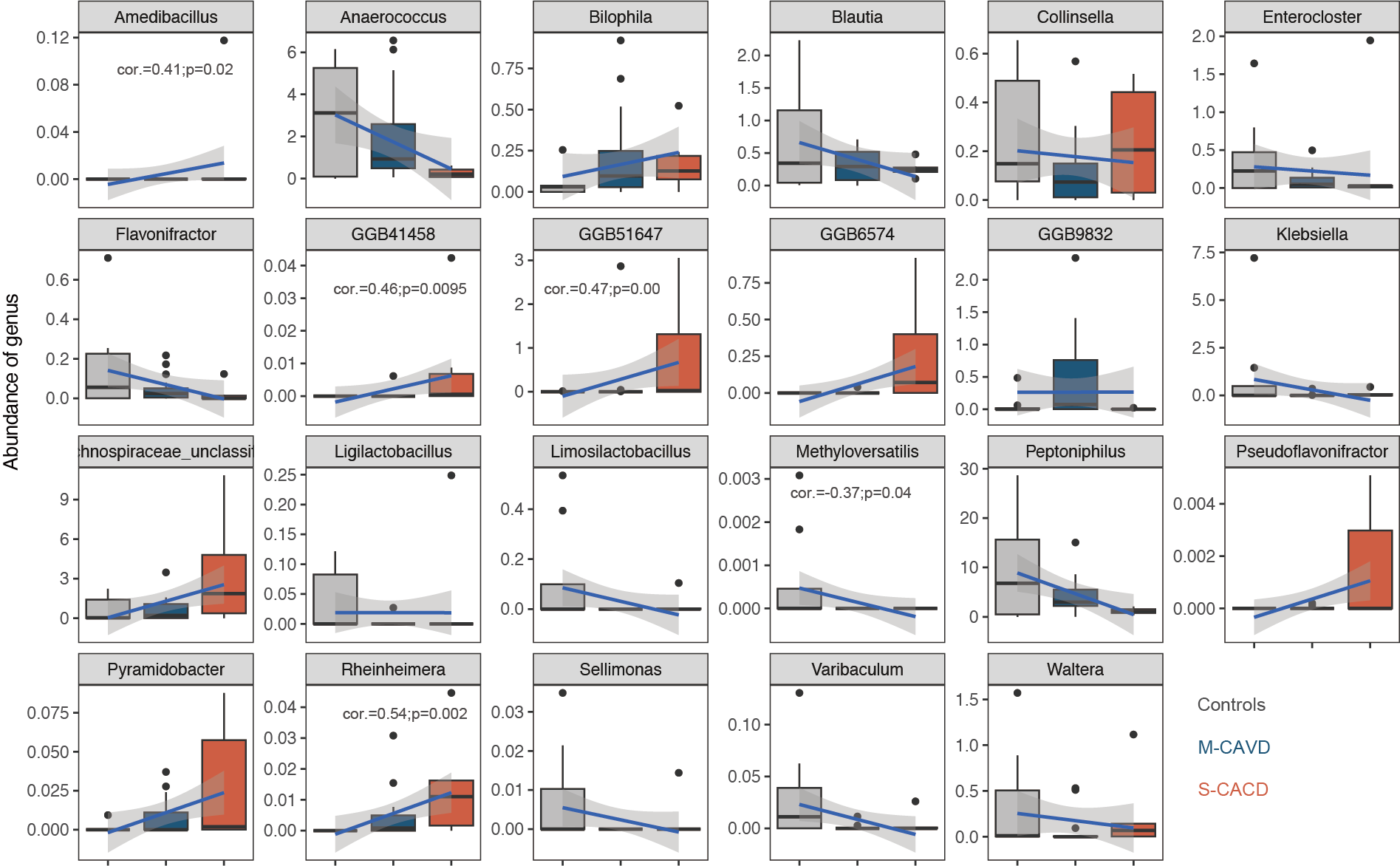


**Figure S3. The spearman correlation analysis identified 23 potential differentially abundant genera associated with disease severity, with significant correlations (p < 0.05) indicated on the figure.** Additional information on other genera is provided in Table S1. The box represents the interquartile range (IQR) of the fold changes, with the line inside the box indicating the median value. Whiskers extend to the maximum and minimum values within 1.5 times the IQR from the quartiles, while points outside this range are plotted as individual outliers. The sample sizes (n) for each group are as follows: Controls (n = 8), m-CAVD (n = 16), and S-CAVD (n = 6). P-values were calculated using a t-test as implemented in cor.test to evaluate the significance of the correlation coefficient.

**Figure S4.**


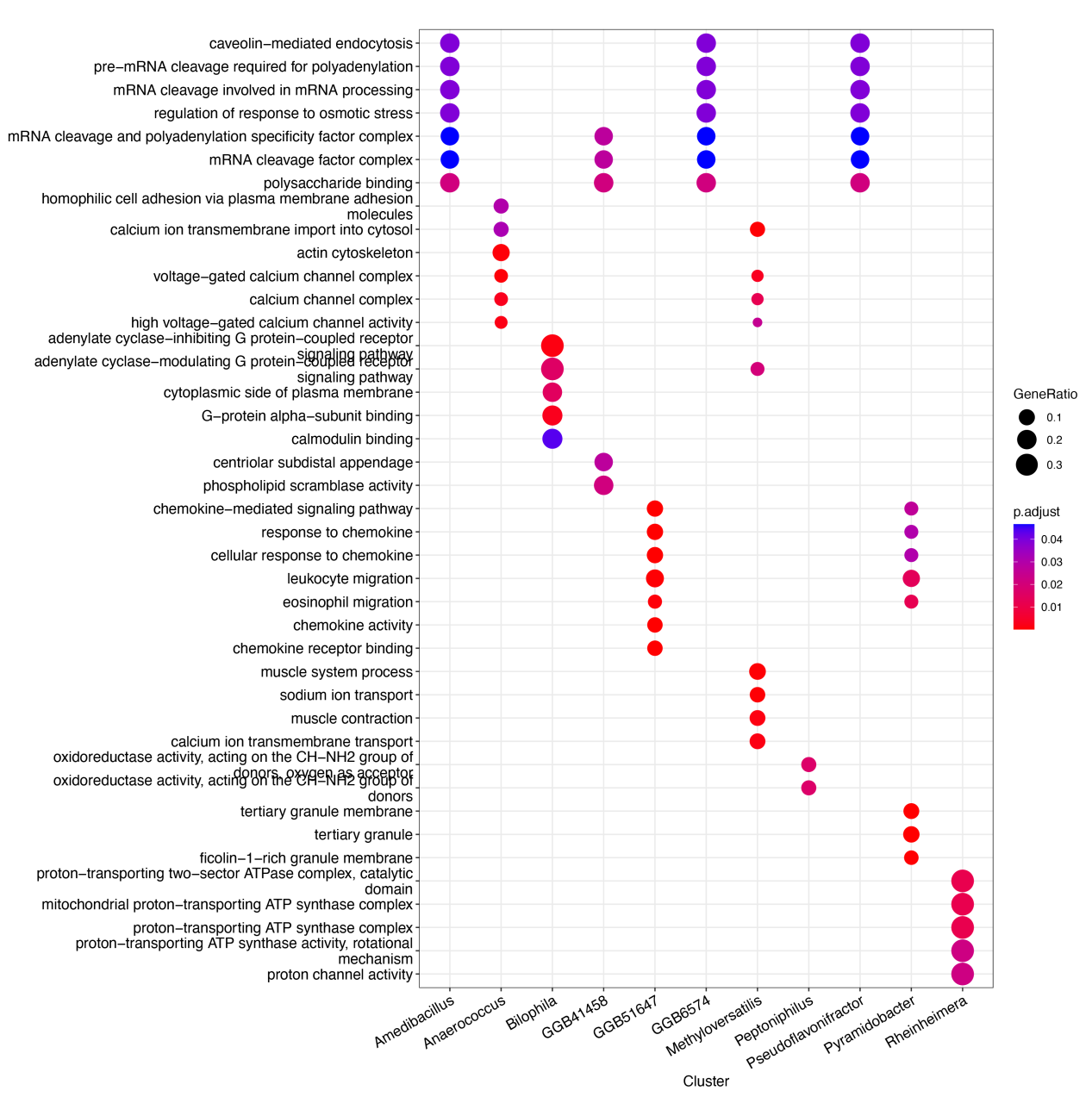


**Figure S4. We conducted an enrichment analysis of differentially expressed genes (P < 0.05, |logFC| > 0.5).** These genes are associated with each genus based on a significance threshold of P < 0.01 and |correlation| > 0.2. P-values for genes were calculated using a negative binomial model with the Wald test as implemented in DESeq2 based on input read counts, and adjusted p-values were computed using the Benjamini-Hochberg method to control the false discovery rate (FDR). P-values for GO enrichment were calculated using a hypergeometric test to assess the over-representation of gene ontology terms, and adjusted p-values were computed using the Benjamini-Hochberg method.

**Table S1. The spearman correlation analysis identified 23 potential differentially abundant genera associated with disease severity.**

| pvalue | spearman_cor. | Genus |
| --- | --- | --- |
| 0.265148487 | -0.21008943 | Ligilactobacillus |
| 0.19393232 | -0.243933489 | Varibaculum |
| 0.147346539 | -0.271079936 | Sellimonas |
| 0.52969979 | -0.119403345 | Limosilactobacillus |
| 0.365309334 | -0.171336905 | Enterocloster |
| 0.041543199 | -0.374351826 | Methyloversatilis |
| 0.631532337 | -0.091251685 | Waltera |
| 0.095913263 | -0.309619657 | Peptoniphilus |
| 0.554586826 | -0.112313363 | Collinsella |
| 0.695248535 | -0.074591797 | Blautia |
| 0.200046437 | -0.24072453 | Flavonifractor |
| 0.91783896 | 0.019667593 | Klebsiella |
| 0.002009015 | 0.541289574 | Rheinheimera |
| 0.090455888 | -0.314561459 | Anaerococcus |
| 0.008067085 | 0.47449367 | GGB6574 |
| 0.064280565 | 0.342067178 | Pseudoflavonifractor |
| 0.094728393 | 0.310674121 | Pyramidobacter |
| 0.276178956 | 0.205411634 | Lachnospiraceae_unclassified |
| 0.059525146 | 0.347970009 | Bilophila |
| 0.081727857 | 0.322959054 | GGB51647 |
| 0.812925153 | 0.045101408 | GGB9832 |
| 0.025060377 | 0.408383811 | Amedibacillus |
| 0.009559015 | 0.465360808 | GGB41458 |

**Table S2. Clinical characteristics of study population (n=31)**

| Characteristic | non-CAVD(n=8) | m-CAVD(n=16) | s-CAVD(n=7) | P*-*value |
| --- | --- | --- | --- | --- |
| Age (year), mean ± sd | 51.7±8.86 | 59.75±8.02 | 60.43±10.00 | 0.087 |
| [Gender](javascript:;) (male), n (%) | 6 (75.00) | 14 (87.50) | 3 (42.86) | 0.074 |
| Hyperlipemia, n (%) | 3 (37.50) | 3 (18.75) | 0 (0.00) | 0.210 |
| Hypertension, n (%) | 5 (62.50) | 4 (25.00) | 4 (57.14) | 0.138 |
| Diabetes mellitus, n (%) | 2 (25.00) | 1 (6.25) | 2 (28.57) | 0.236 |
| Smoker, n (%) | 5 (62.50) | 10 (62.50) | 3 (42.58) | 0.714 |
| Bicuspid aortic valve, n (%) | 0 (0.00) | 5 (31.25) | 2 (28.57) | 0.203 |
